# A novel, bottom-up approach to promote evidence-based HIV prevention for people who inject drugs in Ukraine: protocol for the MICT (‘Bridge’) HIV prevention exchange project

**DOI:** 10.1186/1748-5908-9-18

**Published:** 2014-02-04

**Authors:** Jill Owczarzak, Olga Filippova, Sarah D Phillips

**Affiliations:** 1 Department of Health, Behavior, and Society, Johns Hopkins Bloomberg School of Public Health, 624 N. Broadway, Hampton House Room 739, Baltimore 21205-1996, Maryland; 2 Department of Sociology, V.N. Karazin Kharkiv National University, 6, Svobody Sq. Office 351, Kharkiv 61000, Ukraine; 3 Department of Anthropology, Indiana University, Student Building 130, 701 E. Kirkwood Avenue, Bloomington, IN 47405-7100, USA

**Keywords:** HIV prevention, People who inject drugs, Capacity-building, Nongovernmental organizations, Ukraine, Evidence-based interventions, Implementation

## Abstract

**Background:**

Ukraine has one of the most severe HIV epidemics in Eastern Europe, with an estimated 1.6% of the adult population living with the virus. Injection drug use accounts for 36% of new HIV cases. Nongovernmental organizations in Ukraine have little experience with effective, theory-based behavioral risk reduction interventions necessary to reduce the scope of the HIV epidemic among Ukrainians who inject drugs. This study seeks to promote the use of evidence-based HIV prevention strategies among Ukrainian organizations working with drug users.

**Methods/design:**

This study combines qualitative and quantitative methods to explore a model of HIV prevention intervention development and implementation that disseminates common factors of effective behavioral risk reduction interventions and enables service providers to develop programs that reflect their specific organizational contexts. Eight agencies, located in regions of Ukraine with the highest HIV and drug use rates and selected to represent key organizational context criteria (*e.g*., agency size, target population, experience with HIV prevention), will be taught common factors as the basis for intervention development. We will use qualitative methods, including interviews and observations, to document the process of intervention development and implementation at each agency. Using risk assessments with intervention participants, we will also assess intervention effectiveness.

The primary outcome analyses will determine the extent to which agencies develop and implement an intervention for drug users that incorporates common factors of effective behavioral interventions. Effectiveness analyses will be conducted, and effect size of each intervention will be compared to that of published HIV prevention interventions for drug users with demonstrated effectiveness. This study will explore the role of organizational context on intervention development and implementation, including resource allocation decisions, problem-solving around intervention development, and barriers and facilitators to inclusion of common factors and delivery of a high quality intervention.

**Discussion:**

This innovative approach to HIV prevention science dissemination and intervention development draws on providers’ ability to quickly develop innovative programs and reach populations in greatest need of services. It has the potential to enhance providers’ ability to use HIV prevention science to develop sustainable interventions in response to a rapidly changing epidemic.

## Background

### HIV among people who inject drugs (PWID) in Ukraine

In Ukraine, annual HIV diagnoses have more than doubled each year since 2001 [1,2]. Injection drug use accounts for 36% of new HIV cases, and the majority of HIV infection occurs among PWID and their sex partners [3,4]. The unique drug use practices among Ukraine PWID and their associated HIV risks require tailored, context-specific prevention interventions. In Ukraine, locally produced opiates are often obtained in liquefied form either in pre-loaded syringes, or from a common container. Drugs are often distributed by frontloading or backloading the solution from the dealer’s syringe into the users’ syringes [3]. Loading from common syringes contributes to HIV risk because up to 12 unrelated PWID may load their syringes from the dealer’s same equipment, and the drug solution may be contaminated and passed to other users. In addition, Ukrainian PWID typically inject in groups of network members, and share both drug solution and needles/syringes [5]. Needle exchange programs are unlikely to decrease the indirect sharing that occurs as a result of the prevalence opiates in liquefied form [6]. Current evidence-based interventions (EBIs) for drug users do not specifically incorporate information about these unique injection drug use risks and would require significant tailoring to increase their relevance for Ukrainian PWID.

### The role of nongovernmental organizations in HIV prevention

Nongovernmental organizations (NGOs) are the primary providers of HIV prevention services for PWID in Ukraine. Historically, NGOs initiated the response to the AIDS epidemic, and they have remained the primary providers of HIV prevention services and programs [7]. NGOs are able to promote local involvement, develop and implement low-cost programs, and adapt and innovate in response to changing conditions at the local level [8]. NGOs also have the ability to reach the most stigmatized, marginalized, and at-risk populations, such as PWID and their sex partners, commercial sex workers, and men who have sex with men [7]. In Eastern Europe, these groups are often ignored or misunderstood by governmental HIV prevention initiatives, and are distrustful of governmental programs and workers [9]. Currently, NGOs primarily conduct needle exchange programs, one-on-one direct outreach, peer education, and AIDS information dissemination [9]. While provision of sterile injection equipment is important and has been shown to reduce new HIV infections among PWID [10,11], such programs alone do not address sexual HIV risk among drug users [12]. Moreover, although education efforts among Ukrainian PWID have resulted in high levels of knowledge about HIV transmission and how to reduce risk of infection, it has not reduced their HIV infection rates [4].

### Barriers to adoption and implementation of EBIs

The capacity of NGOs to implement resource-intensive, theoretically informed interventions presents a significant barrier to widespread incorporation of evidence-based interventions into local programming [13]. EBIs often require a high degree of structure and were developed and tested in much more controlled settings than exist in most community organizations [14]. NGO-based providers commonly work with clients who are much more variable than research study participants in terms of age, socioeconomic status, racial/ethnic background, risk behaviors, and gender [15]. Volunteer or low-paid staff without experience implementing evidence-based interventions, high staff turnover rates, small budgets, and few resources combine to create a challenge to sustainable implementation of externally-developed programs [7,9,16,17]. Eastern European HIV prevention NGOs in particular often operate with mean annual budgets of $55,000 in cities with populations over 1 million people [9]. In Ukraine, the national HIV program is significantly underfunded, resulting in lack of financial support for NGOs working with populations most at-risk for HIV, including drug users [18,19].

### The politics of public health policy transfer

When public health policies and interventions are transferred from the US and other Western countries to new contexts (particularly low and middle income countries), they are often met with strong resistance by local experts who see them as undermining their professional training, locally-generated solutions, and context-appropriate programs [20]. They may also resist new programs and services that they see as inconsistent with strongly held values [21]. Moreover, service providers and NGOs are often ambivalent about research and its implications for their specific practices [22,23]. Providers and clients may view the results of research as irrelevant to their needs if results do not reflect their experience-generated knowledge, values, or priorities, and if researchers alone define its focus and significance [24]. Providers consider the relative advantage of the new program over existing services, funding and service obligations, and resource levels in decisions to implement new interventions [25]. Top-down dissemination of researcher-developed interventions undermines this process and creates resistance to uptake of these programs; as a result, community-based service providers may prefer their ‘homegrown’ programs over pre-packaged interventions [26,27].

### Persistent research-to-practice gap in EBI implementation

Initiatives to disseminate EBIs have focused on helping service providers implement programs with fidelity to core elements [14,28,29]. ‘Core elements’ are integral components of the intervention thought to be responsible for its effectiveness and that must be retained in order for HIV risk reduction to occur [18,30,31]. During training, agencies that plan to implement the intervention are instructed that core elements cannot be ignored, added to, or changed. However, service providers rarely implement EBIs with fidelity, despite significant investment of financial, human, and material resources into dissemination efforts. Frontline service providers frequently expand interventions to new populations, include most but not all core elements, or reinvent interventions by combining them with other programs [32-35]. These changes potentially render the intervention ineffective [32,36]. Agencies that participate in capacity-building programs continue to experience high rates of staff turnover, lack access to technical assistance resources, inconsistently evaluate and implement programs, struggle to recruit and retain intervention participants, and operate with limited resources while attempting to implement costly and complex interventions [13,37-39]. At the same time, funding and capacity-building agencies, in partnership with service providers, spend significant resources taking interventions developed through research for one population and adapting and modifying them to fit new target populations [40]. The impact of this frontline ‘tailoring’ process on intervention effectiveness, however, is not well-researched. This process also creates the need to continually adapt each intervention for each new population, requiring significant resources and long-term, unsustainable researcher-agency-funder partnerships. In response to these implementation shortfalls, dissemination researchers have called for more attention to the consequences of implementation fidelity versus adaptation, and models to promote implementation fidelity among frontline service providers have proliferated [41,42].

### Building better interventions through common factors dissemination

From a dissemination and implementation perspective, core elements are often purely theoretical and not easily translatable to practice; do not capture critical aspects of the intervention; and lack specificity to guide service providers in program delivery [43,44]. In contrast, common factors are broader constructs that support behavior change and are incorporated into a variety of EBIs. Generally, effective evidence-based prevention strategies are based on the idea that behavior change requires opportunities and practice, and that change occurs over time [45]. Successful HIV prevention interventions include a framework to understand the HIV risk behavior and change; cognitive, affective, and behavioral skill-building; fostering sustainable social support; tailored, behavior-specific content; and addressing environmental barriers to behavior change [45]. Factors common to effective behavioral interventions can be categorized into three domains: implementation, content, and pedagogy. (See Table 1).

**Table 1:** **Common factors of effective behavioral interventions **[46-53]

**Implementation**	**Content**	**Pedagogy**
Multi-session	HIV/AIDS information	Peer group discussion or interaction
Small group format	Risk Identification and Skill-building to address risk	Demonstration, modeling, role-playing
Knowledgeable, skilled facilitators	Create supportive environment	Tailored, culturally relevant information

### Organizational factors that affect implementation

Organizational, provider, and program characteristics influence the type of intervention an agency develops, and may hinder or facilitate intervention implementation. At the organizational level, factors associated with intervention development include leadership, organizational commitment to and support of the new program, decision-making processes (*e.g*., participatory versus autocratic), stability and adequacy of resources, shared vision and goals within the organization, willingness to initiate change, and dedicated staff to implement the intervention [54-57]. Provider characteristics (*e.g*., demographic, educational) are also associated with intervention adoption and implementation. In addition, intervention adoption and implementation is linked with the intervention’s appeal to providers’ sense of what will work with their specific populations; belief in the intervention’s supporting evidence; willingness to follow either organizational or funding requirements to implement specific programs; and openness to change [58,59]. In addition, programs that could be implemented by small numbers of part-time and unpaid staff with few resources (time, money, space, materials) are more likely to be adopted and implemented [60,61].

### Research methods

#### Study objectives

The MICT study will explore the extent to which NGOs in Ukraine can develop an HIV prevention intervention based on common factors of effective behavioral interventions, and whether these agency-developed interventions reduce participants’ drug use and sexual HIV risk behaviors. We expect that service provider characteristics, including agency size and resources, mission and goals, commitment to intervention development, and staff expertise will affect both intervention development and implementation. Organizations will take these factors into consideration in decisions regarding intervention development, including specific content, target population, number of sessions, and pedagogy. These factors will influence organizations’ ability to implement the interventions they develop as they modify the intervention in response to unanticipated barriers and problem-solving to overcome them.

### Study design

This study combines qualitative and quantitative methods to determine whether Ukrainian NGOs working with injection drug users can successfully develop and implement HIV prevention interventions based on common factors of effective behavioral interventions. We will teach staff from eight Ukrainian NGOs common factors of effective behavioral interventions, theoretical concepts identified as important for behavior change, and key aspects of intervention development. We will document the process of intervention development and implementation at each agency. Then, we will document intervention implementation to assess inclusion of common factors; aspects of program delivery, including reach and scope; and effectiveness.

### Study sites

We recruited eight HIV prevention organizations that work with people who inject drugs from regions with the highest HIV prevalence rates, specifically the eastern and southern regions and the central region [18]. We purposely recruited NGOs to reflect real-world variability in terms of agency history, size, mission and context. Study agencies include at least one of each of the following: an HIV prevention-specific NGO; an IDU-focused NGO that primarily works through street-level outreach and information dissemination; a medically-oriented drug treatment organization; a small NGO that is primarily volunteer-staffed; and a rights-based NGO that advocates for improved policies toward and better treatment services for drug users. The differences between these agency contexts will be a key consideration in final analyses of intervention development, implementation and effectiveness.

### Data collection

Data collection will occur in 4 phases (Baseline, Intervention Development, Intervention Implementation, Effectiveness). (See Table 2).

**Table 2:** MICT study design and data collection phases

**Phase**	**Year**	**Data collection**	**Purpose**	**Source**	**Total number**
Baseline	1	Provider questionnaire	Assess quantifiable aspects of organizational context	Agency director	n = 8
Baseline	1	In-depth interviews	Assess qualitative dimensions of organizational context	Agency director, NGO staff	n = 32 (4/site x 8 sites)
Baseline	1	Organizational histories	Document organization origins, mission, target population, changes over time	Agency print materials	n = 8
Baseline	1	Week-long, face-to-face workshop	Teach NGO staff common factors and train them in intervention development	Location: Ukraine	N/A
Development	1 & 2	1-month interviews	Document intervention development, provide consultation as needed	Agency staff	n = 48 (1/site x 8 sites x 6/year)
Development	2	Intervention manual	Assess common factors, clear goals, well-defined population	Each agency	n = 8
Development	2	Manual checklist	Determine if interventions include common factors, clear goals	Each agency	n = 8
Development	3	3-month interviews	Documentation of intervention development; consultation	Agency staff	n = 32
Implement	2 & 3	Intervention observations	Determine quality of intervention implementation	Video recorded sessions	1 complete intervention/site
Implement	3	Common factors checklist	Assess presence or absence of common factors in implementation	Checklist of observation	n = 8
Implement	3 & 4	Follow-up interviews	Document why common factors included or not	Intervention facilitators	n = 16 (1-2 facilitators/site)
Effectiveness	3 & 4	Baseline & 3-month assessments	Determine whether interventions reduce participants’ risk	Intervention participants	n = 520 (130/site x 4 sites)

#### Phase 1 (Baseline)

To assess agency characteristics that potentially affect intervention implementation, NGO directors will complete a provider questionnaire that will assess organizational capacity and other aspects of implementation context, including number of full-time, part-time, and volunteer staff; staff turnover; types of HIV prevention services provided; client characteristics; and HIV prevention funding sources. In-depth interviews with agency staff will assess non-quantifiable aspects of implementation context and gain additional perspectives on the agency and HIV prevention among Ukrainian NGOs, including experiences implementing evidence-based interventions, sense of preparedness to implement a new program, training experience, and anticipated barriers and facilitators to implementation. Interviews also will focus on the history of the NGO; current and future agency goals and scope of HIV prevention activities; and the interviewee’s personal history of involvement in HIV prevention activities, including his/her tenure in his/her present position, experience with the target population, and views on PWID and HIV risk and prevention strategies [13].

#### Phase 2 (Intervention development)

During Study Years 1 and 2, following training we will conduct regular interviews with NGO staff involved in intervention development. These interviews will focus on decision-making processes regarding intervention development, such as how intervention design is determined; capacity-building activities the agency has conducted to prepare for implementation (*e.g*., additional staff training or recruitment); development of procedures and relationships to facilitate intervention implementation (*e.g*., cooperative agreements with partner agencies, participant recruitment strategies); and barriers to program development. At the conclusion of Study Year 2, we will collect each agency’s intervention manual to document whether an agency’s intervention includes an identifiable target population, clear objectives, and common factors of successful interventions.

#### Phase 3 (Intervention implementation)

During Study Year 2, we will conduct intervention observations of a complete intervention cycle at each participating NGO. Data collected from the video recording will be used to complete a Common Factors Checklist for each session at each site that will record whether the intervention includes, somewhat includes, or does not include common factors of effective behavioral interventions. Two Common Factors Inclusion Scores (quantitative and qualitative) will be calculated for each intervention implemented at each agency. The first score (quantitative) will use a 2-point scale to capture the presence (1 = included) or absence (0 = not included) for each common factor in the implemented intervention. The second score (qualitative) will use a 4-point scale to capture the quality of implementation of common factors. For example, facilitator skills such as ‘subject matter expertise’ will be scored as: 0 = no expertise; 1 = little expertise; 2 = some expertise; 3 = adequate expertise; 4 = expert. These two measures will then be summed and rank ordered separately, and an overall ranking will be calculated for each agency based on these two scores. The four agencies with the highest average ranking will be included in effectiveness component of the study (Phase 4, described below). We will conduct analyses of internal validity (Cronbach’s alpha) for each scale included in the Common Factors Checklist.

We will conduct follow-up interviews with facilitators from each site. During the interviews, each facilitator will be asked questions related to specific elements of the intervention, and about their motivations for including, modifying or eliminating common factors, as indicated by the Checklist. Facilitators also will be asked about agency-level support (financial, personnel, ideological) for continued implementation, evaluation activities, perceived ability to sustain the intervention, number of clients served, issues of client recruitment and retention, and impressions of client satisfaction. Follow-up interviews also will assess quantifiable aspects of intervention implementation, including dosage (contact time with participants and number of sessions) and reach (number and type of participants served).

#### Phase 4 (Effectiveness)

To determine intervention effectiveness at each site, during Study Years 3 and 4, we will conduct baseline and 3-month risk assessments with intervention participants enrolled in the interventions at the four agencies with the highest ranked Common Factors Inclusion Scores. Intervention participants will be invited to participate in this component of the study at the time they are recruited by the agency to participate in the intervention; all intervention participants who are 18 years of age or older will be eligible. A total of 130 participants will complete the risk behavior assessment at each of the four sites (n = 520).

The assessment instrument will be based on the Risk Behavior Assessment (RBA)*;* it will be modified slightly based on the drug use patterns of IDUs in Ukraine. The RBA assesses demographics, health history, drug use, and injection and sex-related risk behaviors (frequency, quantity and duration of use of various drugs including frequency of injecting drugs; injected opiates or an opiate sedative mix or stimulants; injection with network members; used a front or backloading method; drew drugs from a common container; injected with a needle/syringe after other IDUs; number of times had anal, oral or vaginal sex with and without a condom in the past months, had sex with primary and casual partners, and exchanged sex for money or drugs with or without a condom; and number of sex partners in past three months, and same sex partners and partners who use drugs). We will assess psychosocial constructs believed to mediate or moderate risky behaviors. HIV risk reduction behavioral intentions will be evaluated using 7 items on a 6-point scale assessing strength of intentions to reduce HIV risk behavior (alpha = 0.82). HIV-related vulnerability beliefs will be evaluated using 10 items scored on 7-point scales with possible responses from ‘very unlikely’ to ‘very likely’ (alpha = 0.75). HIV Risk Reduction Skills will be assessed by asking participants how many times or how often in the past three months they engaged in HIV preventive behaviors. We will measure HIV-preventive behavioral skill self-efficacy using the self-efficacy scale used in the NIMH Multisite HIV Prevention Trial. Questions about participants’ experiences in the intervention will address benefits of participation, perceived relevance, whether they would recommend to peers, and overall experiences.

### Outcomes

Primary qualitative analyses will examine the relationship between variations in intervention development, delivery and effectiveness at each NGO and qualitative data regarding these service provider characteristics. These analyses will also compare and contrast agencies with different Common Factors Inclusion Scores and intervention effectiveness with respect to similarities and differences in the context of intervention implementation.

ACASI risk behavior assessments of intervention participants at baseline and three-month follow-up will be combined with agency-intervention characteristics (including Common Factors scores) in a hierarchical (mixed-effect) repeated measures analysis of changes in risk behavior pre- and post-intervention. We will use the software program GLIMMIX (v9.2, SAS Institute) — generalized linear mixed models — to test whether interventions can reduce participants’ drug use and sexual HIV-risk behaviors and to explore the influence of intervention and agency characteristics on any reduction in risk behavior. Risk assessments will be analyzed to determine whether or not participants reported decreased injection-risk behaviors in the past month and number of acts of unprotected (without condoms) vaginal or anal intercourse in the past three months. We will fit a hierarchical Poisson regression model to examine whether the intervention can reduce the number of risky drug injections in the past month and number of unprotected vaginal or anal intercourse acts in the past three months. Because the number of risky acts can be very much skewed, the assumptions for the standard linear regression model are often invalid. Behavior assessment as a binary outcome will be analyzed using a hierarchical logistic regression model. Each model will include assessment period (baseline = pre and follow-up = post), a random effect for repeated within-subject assessments, and test covariates. The test covariates include intervention factors (*e.g*., Common Factors score), provider factors (*e.g*., capacity), and participants’ characteristics (*e.g*., demographics, baseline risk-related constructs). Interaction terms between selected covariates and assessment points will be added to these models to test whether covariates are associated with change in risk behavior. If an interaction effect between assessment point and a provider factor is found, subsequent exploratory analyses will examine the influence of various provider factors. Direct tests of the influence of participant-level covariates will be conducted by entering these variables in the last step of the regression. We will conduct separate analyses for each of the four different agency interventions in the effectiveness studies. To determine the feasibility of this approach to intervention development, our analysis will focus on whether these interventions reduce risk behavior. Recruitment of 130 participants by each agency with an 88% retention provides 0.8 power with 0.05 alpha to detect changes (effects), similar to other interventions, of 27% decrease in number of risky drug injections in the past month, and a 24% decrease in percentage having sexual intercourse without condoms in the past three months.

We will use qualitative and quantitative methods to identify agency characteristics, including organizational barriers and facilitators to inclusion of common factors and delivery of a high quality intervention, and implementation context factors, including funding, political and cultural factors that shape intervention development and implementation. Particular attention will be paid to why facilitators and/or agency directors include or eliminate common factors, and the organizational context of intervention implementation, including current and future agency goals, scope of HIV prevention activities, and motivations for developing that particular intervention [17,23,30,62].

### Ethical considerations

This study is approved by the Institutional Review Board at the Johns Hopkins School of Public Health and the Ethics Committee of the Sociological Association of Ukraine. Informed consent is obtained from all participants, including agency staff and clients. All data collection and reporting will be compliant with US and Ukrainian privacy laws, and no report will identify individual participants or study agencies. Data will be securely stored at Johns Hopkins University, Kharkiv University, and the Medical College of Wisconsin. All study records and documents will be stored for no longer than 10 years after the study has ended.

### Trial status

This study began in November 2013 with the recruitment of eight agencies. In May and June 2013, we completed the baseline qualitative component of the study, including organizational assessment interviews, site visits to each agency, and in-depth interviews with staff and directors at each agency. In June 2013, we conducted a multi-day training workshop with agency representatives. In the fall of 2013, study agencies are developing and manualizing their interventions. In spring of 2014, video-recorded agency interventions will be reviewed to determine whether they include common factors of effective behavioral interventions. The agencies whose interventions best reflect the standards of effective behavioral interventions will enter the second phase of the study in July 2014 and participate in intervention evaluation to determine each program’s effectiveness at reducing participants’ drug use- and sex-related HIV risk. This phase will continue through May 2016.

## Discussion

### Strengths and limitations

This study will use both qualitative and quantitative methods to explore intervention development processes, quality of implementation, and organizational factors that affect provider decisions in intervention development and implementation. Direct observation (in addition to self-report) of intervention delivery will provide more accurate documentation of the extent to which common factors are incorporated into each agency’s intervention. Direct observation will enable subsequent analysis of these common factors within the context of the intervention as a whole and capture important aspects of intervention delivery, such as facilitator/participant interactions. Second, qualitative interviews can more fully capture planning processes, barriers and constraints to intervention development and implementation, and problem-solving strategies to overcome these barriers than can more quantitative assessments. Third, the questions this study attempts to answer regarding the role of organizational context on intervention development and delivery are not easily quantifiable and are better suited to qualitative methods. Due to the intensive nature of qualitative research, we designed this study to compare a small number of NGOs (eight), sampled for diversity and strategically selected to reflect key organizational context criteria, including agency size, target population, and experience with HIV prevention interventions. Standard analyses for intervention-control experiments rely on the use of randomization to ensure that intervention and comparison groups are comparable with respect to factors known and unknown that might impact study outcomes. An effectiveness component based on a randomized controlled trial (RCT) design is not appropriate for several reasons. First, randomization is not accepted by the frontline service providers with which we work because they believe that all people who desire to participate in the intervention should receive it, and research participants themselves may not want to be randomized [63]. Moreover, many of the assumptions required of an RCT (*e.g*., participants receive treatment as intended, attrition does not occur) would likely not be met given the realities of study participants’ lives (*e.g.*, housing instability, dropout due to incarceration or drug use). In addition, it is likely that each NGO’s intervention will be unique in reflection of the different contexts in which it is developed. To be useful, the results of research must be externally valid — relevant to the population they intend to reach [64]. Given the aims of this study — to give frontline service providers the tools to build interventions that will maximally benefit their populations and reflect their unique contexts — external validity was given priority in study design consideration. Because it is a naturalistic study of the effectiveness of an intervention as implemented by frontline service providers, we cannot control for all conceivable threats to internal validity. Previous research has demonstrated that the effects of treatment in observational and in RCTs were similar in most areas, and therefore produce valid information [65]. Therefore, we will calculate the effect size at each of the agencies to determine whether the observed levels of pre-/post-intervention risk reduction are comparable to those of other behavior change interventions for drug users.

### Significance

This study proposes a model of intervention development that provides NGO staff with the tools to develop their own evidence-based prevention programs by teaching them ‘common factors’ of successful HIV prevention programs. This strategy can potentially increase the capacity of HIV prevention NGOs to conduct theory-based, multi-session risk reduction interventions [28]. If this study demonstrates the feasibility of disseminating the common factors of effective behavior change interventions to frontline service providers, we anticipate several opportunities to broaden its impact. First, it could lead to the creation of training modules that emphasize skill-building and organizational capacity around behavioral risk reduction strategies more generally, rather than similar trainings based on specific interventions that must be significantly modified for new contexts. Second, this proposed model of intervention development could shift the ways in which local HIV prevention service providers are trained. By providing local experts with the tools of prevention intervention development based on principles of effective behavioral risk reductions, this model addresses the shortcomings of current dissemination methods and draws on the knowledge and experience of frontline service providers as a key resource in fighting the HIV epidemic.

## Abbreviations

NGO: Nongovernmental Organization; HIV: Human immunodeficiency virus; PWID: People who inject drugs; EBI: Evidence-based intervention; RBA: Risk behavior assessment; CDC: Centers for disease control and prevention.

## Competing interests

The Authors declare that they have no competing interests.

## Authors’ contributions

JO conceptualized the study and detailed the main content of the paper. JO leads the study and is responsible for all implementation aspects of the study, and prepared the initial manuscript draft. OF leads the research team in Ukraine and contributes theoretical and methodological expertise to the project. SP contributed to the research design and analysis of the qualitative component of the project. All authors contributed to drafting the manuscript, and all have read and approved the final manuscript.
